# New Patient Referral Patterns May Reflect Gender Biases in Orthopedics

**DOI:** 10.7759/cureus.40935

**Published:** 2023-06-25

**Authors:** Nicholas Bertha, Timothy Visser, Nikkole Haines

**Affiliations:** 1 Department of Orthopaedics, Penn State Health Milton S. Hershey Medical Center, Hershey, USA

**Keywords:** shoulder/elbow arthroplasty, referral pattern, reconstruction hip and knee surgery, total joint arthroplasties, gender bias, gender equity

## Abstract

Background

Orthopedic surgery traditionally has been a male-dominant specialty with the lowest percentage of female residents and female faculty of all medical specialties. Prior studies demonstrate gender biases from both referring providers and patients. This study investigates surgeon, referring provider, and patient demographic differences in new patient orthopedic referrals.

Methodology

A retrospective chart review was performed to analyze the demographics of new patients referred to male and female orthopedic surgeons within adult reconstruction and shoulder/elbow specialties at a single academic institution. Patients and referring provider demographics were compared for male and female orthopedic surgeons. Statistical analysis utilized Student’s t-test and chi-square analyses for quantitative and qualitative data, respectively.

Results

In total, 2,642 new patients were analyzed, with 2,084 patients being referred from a provider, and 306 patients requesting specific providers. When compared to male surgeons, female surgeons had fewer referrals from male providers (45.3% vs. 50.3%, p = 0.03) and no difference from female providers (30.6% vs, 29.9%, p = 0.72). The female adult reconstruction surgeon had fewer internal referrals compared to a male surgeon of similar experience and time at the institution (8.4% vs. 12.8%, p = 0.03). Female patients requested male surgeons more frequently than female surgeons (76.7% vs. 23.3%, p = 0.02).

Conclusions

New patient demographics differed between male and female orthopedic surgeons at a single academic institution with more male referring providers referring to male surgeons. Female patients requesting male orthopedic providers may reflect patient and specialty-driven biases. There remains a need for additional female representation in orthopedic surgery, and new patient referral patterns may be a marker to assess and monitor gender biases.

## Introduction

Orthopedic surgery is historically a male-dominant specialty with the lowest percentage of female residents and female faculty of all medical specialties (about 14% and 6.5%, respectively). Furthermore, despite national increases in female residents and faculty in all specialties, orthopedic surgery shows the slowest change of all specialties [1].

From 1999 to 2009, primary care physician (PCP) referrals to specialists increased by 159% [2]. Current referral patterns do not reflect clinical excellence quality measures, leaving more subjective and nonclinical reasons involved in the referral decision-making process [3]. Referral decisions involve a complex mix of patient, physician, and healthcare system structural characteristics, with considerations also given for professional network, communication abilities, commonality of medical records, and insurance status [4-6]. In addition to these explicit factors influencing referral patterns, there exists the potential for various biases to enter the decision-making process.

Patients have been shown to have biases when evaluating outcomes based on surgeon gender. Sarsons et al. demonstrated that referring physicians view patient outcomes differently depending on the performing surgeon’s gender [7]. For example, female surgeons have a sharper drop in referral rate after a patient death and smaller increases in referrals after good patient outcomes. Sarsons et al. also showed that referring providers who have a bad experience with one female surgeon are less likely to refer to other female surgeons in the same specialty. The same was not true for male surgeons. As a result, gender bias may influence referral patterns and, consequently, the demographics of new patients to male and female orthopedic surgeons. Recent studies have supported these findings in other surgical subspecialties with data showing that male referring providers refer more frequently to male surgeons [8].

Overall, most patients prefer choice in the referral decision process, even if that means longer waiting times [9]. Studies have also shown that when patients have surgeon preferences, these preferences were often related to the surgeon’s age, race, and/or religion [10]. In prior studies primarily including surgical subspecialties other than orthopedics, when female patients had a gender preference, they overwhelmingly preferred a female surgeon, though that option is not always available in every health system or medical specialty [11,12]. Regarding orthopedic surgery, even when female patients had a male orthopedic surgeon preference, it was influenced by prior experiences with orthopedic providers who are overwhelmingly male [10]. On the other hand, some evidence suggests patients may place greater emphasis on the surgeon’s characteristics (including reputation, competency, and interpersonal skills), hospital characteristics and reputation, and word-of-mouth/physician referrals over gender preference [13]. Nonetheless, gender preferences for surgeons exist in both patients who seek orthopedic surgeons and providers who refer patients. The purpose of our study was to investigate whether demographic variables such as the gender of referring physicians and patient preferences were associated with differences in new referral patterns among male and female adult reconstruction and shoulder/elbow orthopedic surgeons.

## Materials and methods

Institutional review board approval was obtained for this study (STUDY00014874) and attention was paid to preserving patient confidentiality. A retrospective chart review was performed to analyze new patient demographics within the adult reconstruction (three male and one female providers) and shoulder/elbow (two male and one female providers) specialties at a single academic institution during the 2019 calendar year. We excluded new patients who were previously seen by another provider in that specialty, new patients from emergency department referrals, patients from prisons, and patients less than 18 years old as these groups have differing patterns of referrals or the inability to adequately select a specific physician. Otherwise, we included all new patients seen above the age of 18.

Chart review of new patients evaluated the referring provider and their gender as well as the demographics of patients who requested to see specific providers. We performed quantitative analysis via Student’s t-test to determine if there was a difference between male and female providers and the corresponding gender of the referring provider. We grouped the referring provider into two groups. The first group was internal providers (providers within their own orthopedic department), and the second group was outside referring providers (any provider outside their orthopedic department). When examining patient requests for surgeons, we performed a qualitative analysis via the chi-square test to compare the expected number of patients who would request male versus female surgeons.

## Results

Between the adult reconstruction service and shoulder/elbow service, there were a total of 2,642 new patients seen in the 2019 calendar year. Of those new patients, 2,084 patients were referred by a provider, and 558 patients were self-referred. We found that female surgeons had fewer referrals from outside male providers (45.3% vs. 50.3%, p = 0.03) without difference in referrals from outside female providers (30.6% vs. 29.9%, p = 0.72) when compared to their male counterparts (Table 1). The female adult reconstruction surgeon also had fewer internal referrals compared to a male surgeon of similar experience and time at the institution (both hired following a fellowship in the same year) (8.4% vs. 12.8%, p = 0.03). A similar comparison could not be made for the shoulder/elbow providers as there was not a pair of providers with similar levels of experience/time at the institution.

**Table 1:** Referrals from providers by gender. When looking at the expected number of consults from referring providers, female physicians saw fewer consults than expected from male referring providers and male physicians saw more consults than expected from male referring providers. Numbers are rounded to the nearest whole person. *: statistically significant

	Actual	Expected	P-value
Female surgeons receiving consults from female providers	254	250	0.72
Male surgeons receiving consults from female providers	542	546	0.72
Female surgeons receiving consults from male providers	376	405	0.02*
Male surgeons receiving consults from male providers	912	883	0.02*

**Figure 1:**
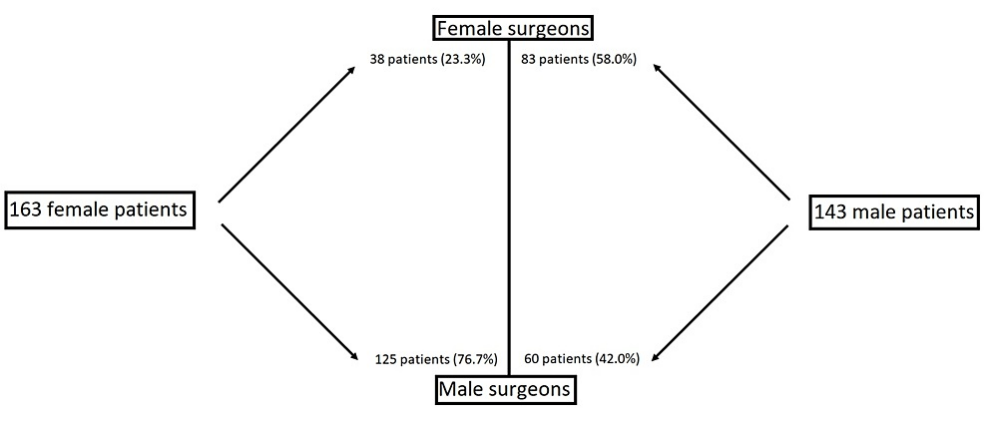
Percentage of patients requesting to see specific providers by patient gender and provider gender. Of the 163 female patients requesting a provider, 76.7% requested a male surgeon. Of the 143 male patients requesting a provider, 42.0% requested a male surgeon.

We found 306 of the 2,642 (11.6%) patients in the study requested a specific provider. Female patients requested fewer female providers (p < 0.01) and more male providers (p < 0.01) than expected. When evaluating male patient physician requests, we found that males requested fewer female providers (p < 0.01) and more male providers (p < 0.01) than expected. When looking at the adult reconstruction department, the female provider received a greater proportion of female patients with a preference for a female provider (p < 0.05) and a lower proportion of male patients with a preference for a female provider (p < 0.05) (Table 2 and Figure 1). No differences were found in the shoulder and elbow department (p > 0.05).

**Table 2:** Patient requests for physicians by gender. When evaluating patient requests for specific providers, both male and female patients requested female providers less than expected and requested male providers more than expected. Numbers are rounded to the nearest whole person. *: statistically significant.

	Actual	Expected	P-value
Female patients requesting female surgeons	38	65	<0.01*
Male patients requesting female surgeons	60	87	<0.01*
Female patients requesting male surgeons	125	99	<0.01*
Male patients requesting male surgeons	83	57	<0.01*

## Discussion

We believe that our study highlights that despite progress in reducing gender bias in medicine [1], this bias continues to persist. We wanted to expand on previous studies that look at gender bias related to patients [14,15] and expand this concept to how important of an issue this is for the providers as well. We felt gender disparity would be most appropriate to evaluate in primarily elective-based specialties such as adult reconstruction and shoulder and elbow as patients often do have a choice in their surgeon. Ideally, this study could have included other subspecialties; however, the disparity in female orthopedic surgeons in some subspecialties at our institution made this impossible to perform highlighting that this issue continues to need to be addressed. Prior studies show that there is a discrepancy in female representation in orthopedics based on geographic region, although there is not a clear cause of this, and it is likely multifactorial [16].

Our hope is that this research will help draw attention to gender bias and disparity within the medical community and among other providers. While this issue is complex and multifactorial, we would hope that this awareness would inspire other institutions to make a concerted effort to not only encourage females to go into orthopedics but also put greater consideration into hiring additional female orthopedic surgeons. While this clearly will not completely solve the issue, the goal would be to reduce the inequality gap. Prior studies have shown that diversity among providers allows patients to be more comfortable and improve the overall patient experience [17,18]. By increasing the diversity of providers, we will gain new perspectives as well as provide patients with a provider that they feel they relate to and who understands their cultural/social background better.

The difference in new patient referral patterns between male and female providers highlights gender bias in medicine and orthopedic surgery (whether implicit or explicit). Despite improved numbers of women in orthopedics, the disparity between male and female providers remains [1,19]. Not only is female representation in orthopedics low but recent studies continue to show disparities in female representation in leadership positions in orthopedics [20]. We feel that these disparities perpetuate conceptions/biases regarding females in the specialty.

It is important to realize that all persons have biases, even in the medical field. New interest has grown in evaluating biases of physicians, and that we develop implicit biases from our culture as well as education [21,22]. Both provider referral patterns and patients’ requests for surgeons show a bias for male orthopedic surgeons. This finding is important as it suggests that biases of surgeons are present both within the medical field and in the view of the public. We believe that further studies evaluating the public perception of orthopedic surgeons are important and may further elucidate information regarding this potential bias. Our hope is that studies like ours can help bring attention to these disparities to promote change moving forward. As suggested by Rohde et al. and others, identifying factors that promote women to choose or not to choose orthopedics and creating better mentorship programs for women in orthopedics may be ways to increase representation in the field [23,24].

One limitation of our study is that it was performed at a single academic institution and this may not be generalized to the entire population. There may be differing patterns at other institutions or in a private practice setting. Additionally, the number of surgeons included in the study was small (we included all surgeons of each subspecialty at our institution) and more surgeons would provide increased power for analysis. This would also help remove underlying bias that could be based on surgeon-specific characteristics, including the level of surgeon experience, practice in a specific geographic area/institution, surgeon technical choices (including utilizing specific approaches, computer-assisted navigation, robotics, etc.), and surgeon reputation that may influence referral patterns. It is hard to determine whether this issue is more significant in the United States where this study was conducted, or if this bias is generalizable to other countries as well. One study examining otolaryngologists in Germany suggested that a higher number of females are entering medical schools, but there is still a disproportionately small number of females in leadership positions in the specialty [25]. More research regarding the generalizability of physician-related gender bias internationally can provide more insight into this problem.

The study size is another limitation of our study. The small study size affected the power of the data, particularly when analyzing patient requests of specific surgeons. This population was much smaller overall and smaller yet for each surgeon. A larger sample size would improve the power of the analysis and provide more insights into differences in patient requests and examine for underlying bias among the general population for male versus female surgeons.

## Conclusions

Overall, our study suggests bias exists among patients and referring providers who favor male adult reconstruction and shoulder/elbow orthopedic surgeons for new patient referrals at a single academic institution. Although the exact decision-making process and reason for the difference in referral patterns are not clearly demonstrated in this study, this study suggests that gender bias may not be isolated to a single institution. Further promotion of female involvement in the orthopedic surgery specialty will continue to promote diversity and help eliminate existing gender biases and improve the overall patient experience.
